# Toward a nanopaper-based and solid phase immunoassay using FRET for the rapid detection of bacteria

**DOI:** 10.1038/s41598-020-71285-3

**Published:** 2020-09-01

**Authors:** Bentolhoda Heli, Abdellah Ajji

**Affiliations:** 3SPack NSERC-Industry Chair, CREPEC, Département de génie Chimique, Polytechnique Montréal, Station Centre-Ville, P.O. box 6079, Montréal, QC H3C3A7 Canada

**Keywords:** Biochemistry, Chemical modification, Biosensors, Design, synthesis and processing, Nanoparticles, Quantum dots

## Abstract

In this study, we propose a novel sensitive solid-based immunosensor developed on a plasmonic nanopaper platform for the detection of *Escherichia coli* (*E. coli*) bacteria. This plasmonic nanopaper that comprises of carboxylated bacterial cellulose (CBC) impregnated with gold nanoparticles (AuNP-CBC), employed as a quencher and a sustainable functionalized platform to be conjugated with protein A. Thus, the conjugated protein A allows the aligned linkage of EAb-QD (anti-*E. coli* conjugated quantum dot) and EAb-AF (anti-*E. coli* conjugated Alexa Fluor 488). Interestingly, once *E. coli* was captured by the AuNP-CBC/EAb-QD or AuNP-CBC/EAb-AF, the energy transfer from the QD or Alexa Fluor fluorophores is triggered due to the conformational change in the antibody structure and this, in turn, causes a decrease in the distance between fluorophores and the quencher nanopaper and, therefore diminishing their photoluminescence. The immunosensors performed successfully to recognize *E. coli* at concentrations as low as 50 CFU mL^−1^ in the standard buffer. The examined functionality of the immunosensors in a real matrix such as chicken extract and lettuce juice demonstrated a highly efficient response while QD is the main fluorophore with a limit of detection around 100 CFU mL^−1^.

## Introduction

During the last decades, the outbreak of foodborne diseases has seriously threatened people lives worldwide and led to a considerable number of hospitalizations and even deaths^1^. Indeed, the incidence of foodborne infection is a result of ingesting food contaminated by foodborne pathogens such as bacteria, viruses, and parasites. To prevent its subsequence, precise monitoring and early recognition of pathogen would be the primary step to be ensured about the safety of our food^2^.

Upon comparing various categories of biosensors developed for pathogen detection, immunosensors have attracted plenty of attention due to their flexibility and compatibility in numerous transducer techniques such as electrochemical (voltammetry, impedancemetry, potentiometry), optical (fluorescence, SPR) and mass-based^3–5^. The excellence of an immunosensor is directly dominated by the affinity binding of the antigen–antibody that this makes it uniquely sensitive and selective toward a specific target^6^. Being fast, selective and sensitive, low-priced, in-situ and user-friendly, immunosensors can fully address the challenges associated with traditional bacteria detection approaches, such as; costly analysis, long processing time, and needs in scientific expertise^7^. Given these benefits, its applications have been widely explored in the environment, medical and, food industries that designed either on a solid-based platform or solution-phase method^8,9^.

Assuredly, the integration of nanomaterials with immunosensing techniques has caused a huge improvement in its functionality and sensitivity^10–12^. Nanoscale materials, with a size less than 100 nm are endowed with unique physical and chemical properties such as; high surface area, optical, and electrical characteristics, which offer a myriad of opportunities in sensing technology^13,14^.

Among the various biosensing approaches and fabricated bioassays, fluorescence-based methods, such as the Förster (fluorescence) resonance energy transfer (FRET), have increasingly emerged as a promising approach due to their sensitivity, selectivity, and rapidness^15^. The feasibility of a FRET-based immunosensor has been studied in a variety of configurations for the detection of the pathogen^16–18^. This phenomenon includes a non-radiative energy transfer between a pair of donor and acceptor molecules, with an extreme sensitivity to their distance^19^. The performance of FRET can be enhanced by maximizing the overlap of the donor emission spectrum and absorbance spectra of the acceptor^20^. Basically, the most frequently used immunosensing based-FRET scenarios consider a non-competitive (e.g., sandwich model and direct interaction)^18^ or competitive interaction^17^.

Owing to their narrow emission spectra, broad absorbance band, and high photostability, semiconductor quantum dots (QDs) have proven to be one of the most effective donor candidates^21^. Particularly, their combination with size-tunable gold nanoparticles (AuNPs), with an efficient fluorescent quenching over a broad range of photoexcited QDs, enables them to be excellent donor–acceptor pairs for bioassay tools^22,23^.

Recently, exploiting the features of bacterial cellulose (BC) (such as crystalline nano-micro fibril structure, high mechanical strength, high porosity, surface area, and transparency) in combination with in-situ synthesized noble metal nanoparticles (gold and silver nanoparticles) has led to the development of improved plasmonic nanopaper^24^. Subsequently, its application as a successful optical sensor of either water-soluble species or volatile organic compounds (VOC) in the dry state has been ascertained^25–27^.

**Scheme 1.**
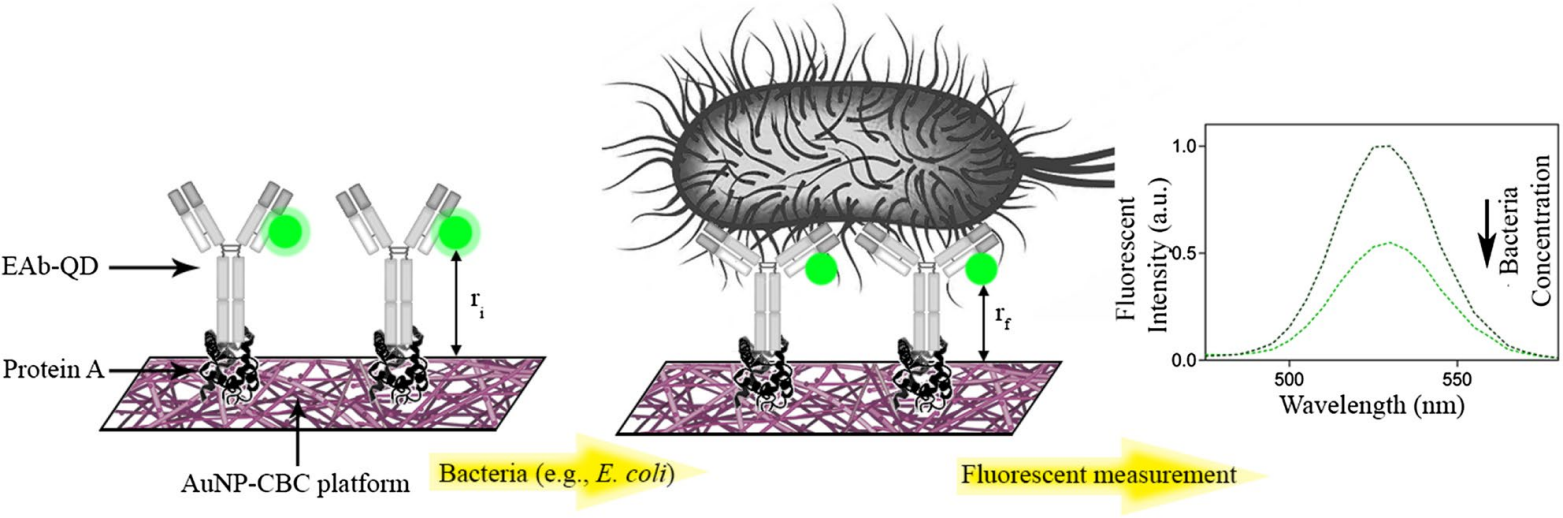
The operational concept of the proposed immunosensor. The carboxylated bacterial cellulose impregnated with AuNP (CBC-AuNP) is implemented as a solid platform to support the essential biorecognition elements, protein A, and labeled antibody with fluorophores. Upon recognizing bacteria, a conformational change occurs in the 3D structure of the EAb-QD that leads to the decrease of QD photoluminescence by the immunosensor. This phenomenon is due to the reduction of effective distance between the donor (QD) and acceptor (AuNP-CBC) from r_i_ to r_f_. It should be noted that the scale of this scheme is irrelevant^30^.

To the best of our knowledge, there is no report of as-synthesized nanopaper applied in a FRET-based immunosensor. In this paper, we describe a novel, versatile, and sensitive immunosensor based on FRET, which utilizes gold nanoparticles embedded in carboxylated BC (AuNP-CBC) as a nanopaper-based acceptor. As Scheme 1 presents the operational concept of the proposed immunosensor, the distinctive structure of the CBC and its abundant functional groups facilitated the layer-by-layer assembly of gold nanoparticles and biomolecules. First, protein A was covalently immobilized on the nanofiber surface of the AuNP-CBC. Second, the anti-*E. coli* antibody conjugated QD (EAb-QD) or the anti-*E. coli* antibody conjugated Alexa Fluor (EAb-AF) bonded with the protein A in a well-oriented configuration. The interaction of the analyte with the labeled antibody caused an alteration in the photoluminescence of fluorophores. This was due to the change of the distance between the fluorophores and the acceptor nanopaper, as a result of the conformational change induced in the antibody structure after its interaction with the analyte^28,29^. The performance of this nanopaper-based, immunosensor was evaluated through the response gathered from the interacting bacteria dispersed in a standard buffer and also in real matrices^30^.

## Experimental methods

### Reagents and instruments

The nanopaper-based substrate, bacterial cellulose (BC), was obtained from Nanonovin Polymer Co. (Mazandaran, Iran). To oxidize BC and create carboxyl groups, Sodium hypochlorite solution (NaOCl), 2,2,6,6-Tetramethyl-1-piperidinyloxy 98% (TEMPO) and sodium bromide (NaBr) were purchased from Sigma-Aldrich Co. LLC (Oakville, Canada). The reagents such as 2-(*N*-Morpholino) ethanesulfonic acid (MES), the tablet of phosphate-buffered saline (PBS), *N*-Ethyl-*N*′-(3-dimethylaminopropyl) carbodiimide hydrochloride (EDC), citric acid trisodium salt and gold (III) chloride trihydrate (HAuCl_4_·3H_2_O) were provided from Sigma-Aldrich. The anti-peroxidase antibody produced in rabbit and horseradish peroxidase (HRP) (peroxidase from horseradish type VI) were acquired from the same supplier. TMB (3,3′,5,5′-Tetramethylbenzidine) solution substrate, used as HRP substrate for the enzymatic reaction, was purchased from Thermo Fisher Scientific Inc. (Ottawa, Canada). Also, Sulfo-NHS (*N*-hydroxysulfosuccinimide) and Pierce recombinant protein A were provided from Thermo Fisher Scientific Inc. The Carboxyl Quantum dot (Qdot 525 ITK) was supplied from Invitrogen Co. (Carlsbad, Canada). Polyclonal antibody produced in rabbits against *E. coli* (DH5α) was purchased from Bioss Inc. (Ma, USA). Also, the conjugated form of the mentioned antibody to Alexa Fluor 488 was provided from the same company, both dispersed in just PBS. The other reagents used in the experiments that are probably not mentioned here were obtained from Sigma-Aldrich.

An Amicon ultra-0.5 mL centrifugal filter, MWCO 100 kD, was acquired from Millipore Corp. (Ontario, Canada). The samples were placed in the Costar 96 microplates (black, transparent, and flat bottomed) provided by Corning Inc. (Kennebunk, Me). The thermo-shaker incubator for microtube and plate rack, used in both conjugation and immobilization, was obtained from MBI (Montreal Biotech Inc., Canada). Measuring UV–Vis absorbance spectra and fluorophores emission spectra were carried out with the Infinite 200 PRO (Tecan, Switzerland) microplate reader. Scanning electron microscopy (SEM) was performed by Hitachi’s ultra-high resolution SU8220 FE-SEM operated at 1 kV (Hitachi High-Technology Cor., Japan).

### Carboxylation of Bacterial Cellulose (CBC)

In general, the carboxyl functional groups are created on the nanofibrils of the BC by the TEMPO-oxidation procedure. As previously described^31^, to obtain the carboxylated BC (CBC), 250 mg of dried BC was dispersed in 150 ml of Milli-Q water, which contained dissolved TEMPO (7.8 mg) and NaBr (78 mg). To start the oxidation, 11 wt% NaOCl with the ratio of 8:1 (mmol/g BC) was added to the solution, and pH was adjusted to 10.5 by adding 0.1 M HCl. Since the pH of the solution decreased during the oxidation reaction, it was kept constant at a pH of 10.5 by adding 0.1 M NaOH. After an hour of gentle mixing at room temperature, the reaction was stopped by adding ethanol to reach pH 7. The CBC was separated from the reaction solution and washed sufficiently with ethanol, and then thrice washed with plenty of Milli-Q water. The obtained CBC was finally preserved in Milli-Q water at 4 °C.

To determine the content of created carboxyl groups on the BC fibers, conductometric titration was performed on the as-oxidized BC^32^. Accordingly, 50 mg of dried and oxidized BC was dispersed in 50 ml of Milli-Q water. Afterward, all carboxyl groups were protonated by adding 0.1 M HCl to reach a pH of around 2.5. Subsequently, the suspension was titrated with 0.01 M NaOH, and the changes in the conductivity of the solution were recorded using the Oakton PC2700 Benchtop Meter. It was finally terminated once pH attained 11. Correspondingly, the carboxyl group content was estimated by drawing the titration curve.

### In-situ synthesis of AuNP within CBC (AuNP-CBC)

The CBC was impregnated with AuNPs in accordance with Turkevich procedure^33^. As such, 15 pieces of CBC (1 × 1 in^2^) were soaked and stirred in 100 ml of the precursor solution, hydrogen tetrachloroaurate (III) hydrate. In order to alter the population density of the fabricated AuNPs, this procedure was carried out individually with various concentrations of precursor solutions: 6, 11, 23, and 45 μM. After bringing the mixture to boiling point, the required citric acid trisodium solution (40 mM) was rapidly added to the container which was 75, 125, 250 and 500 μl, with respect to the precursor concentration. Following the same reaction condition for 1 h, the gold ions were gradually reduced to AuNPs within the nanofibrils of the CBC. The formation of the AuNPs was indicated by the color change of the CBC from colorless to maroon. At that moment, the mixture was stirred without any heating to reach room temperature. Then, the synthesized AuNP-CBCs pieces were separated from the mixture and washed with Milli-Q water to remove all unreacted Au ions and free AuNPs. The resulted AuNP-CBC pieces were dried at room temperature by keeping them between sheets of Teflon film to preserve them from wrinkling.

### Protein A immobilization on the CBC and the AuNP-CBC (CBC-PA and AuNP-CBC-PA)

The protein A was covalently immobilized on the surface of the CBC and the AuNP-CBC through EDC/Sulfo-NHS chemistry. In short, the activated carboxyl groups on the surface of the nanofibrils were bound to the primary amines of the protein A. The immobilization procedure was as follows: The respective nanopapers were cut into circular shapes of 6 mm and placed into a microplate 96 wells. Then, the nanopaper platforms were sequentially washed with water, and 0.01 M MES buffer pH 5 (each step was done for 30 min at room temperature and 650 rpm). After preparing fresh solutions of EDC and Sulfo-NHS in 0.01 M MES pH 5, 40 µl of their mixture, composed of 5 mM EDC and 4.6 mM Sulfo-NHS with the ratio of 1:2 (mole/mole), was added to the nanopaper platforms. The activation was completed in 25 min by incubating in the thermo-shaker at room temperature and 650 rpm.

As the ultimate purpose is to obtain the immobilized protein A on the AuNP-CBC, successive experiments were conducted to determine the efficient conditions of immobilization. These conditions were first evaluated on the surface of the CBC and later inducted into the AuNP-CBC. The strategy was as follows: (1) Investigating the effect of pH: 40 µl of 0.1 mg/ml protein A dispersed in 0.01 M MES with pH 4.1 and PBS with pH 7.4 was separately added to the activated CBC. (2) Determining the optimum amount of the protein A: 40 µl protein A with concentrations of 0.3, 0.1, and 0.033 mg/ml (dispersed in the buffer selected from the earlier experiment) was immobilized on the surface of the activated CBC. The immobilization was accomplished through 2 h incubation in the thermo-shaker at room temperature and 650 rpm. Then, the unreacted protein A was removed and kept for a later Bradford assay analysis. After washing, the resultant CBC-PA with 100 µl MES buffer solution (0.01 M, pH 5), the unreacted active carboxylates were blocked with 50 µl 1% BSA in PBS (0.01 M, pH 7.4) for 1 h at room temperature. The obtained nanopaper complex was then washed with a 50 µl PBS buffer solution and stored at 4 °C for the following experiments.

Hence, the final AuNP-CBC-PA platform was acquired by applying the determined immobilization conditions under similar experimental circumstances.

### Conjugation of antibody to quantum dot

Two different antibodies were explored, anti-HRP (HAb) as a model antibody to evaluate the functionality and validity of CBC-PA and AuNP-CBC-PA and anti-*E. coli* (DH5α) (EAb) to develop the immunoassay platform for pathogen detection. The conjugation procedure was carried out by using QD and antibody with stock solutions of 8 µM and 1 mg/ml to achieve the final concentrations of 60 nM and 16 µg/ml, respectively, following the previously described method^34^. At first, the fresh solution of EDC/Sulfo-NHS, as prepared before, was mixed with 200 µl of QD dispersed in MES buffer (0.01 M, pH 5) with the ratio of 1,000:1, EDC: QD (mole-based). After that, the activation of the QD carboxyl groups was completed by incubating the mixture in the thermo-shaker for 30 min at room temperature and 650 rpm. The excess solution was eliminated through Amicon Ultra-0.5 ml centrifugal filters, cutoff 50 kDa for 7 min at 12,000 rpm (g-force ≈ 13,151). Then HAb or EAb was added to the resulting filtrate and activated QDs redispersed in a 200 µl MES buffer (0.01 M, pH 5). Next, it was incubated for 2 h at 24 °C and 650 rpm. After, the free activated carboxyl groups were blocked by adding 20 µl Tris 20 mM and incubating the mixture for 30 min in the same conditions. The obtained conjugate solution was filtered by Amicon Ultra-0.5 ml centrifugal filters, cutoff 100 kDa. Finally, the resultant Ab-QD conjugate was diluted in PBS buffer (0.01 M, pH 7.4) to reach the as-mentioned concentration and preserved at 4 °C for following manipulations.

### Evaluation of CBC-PA and AuNP-CBC-PA by immobilizing anti-HRP and anti-HRP conjugated QDs

To verify the functionality of CBC-PA and AuNP-CBCs-PA, HAb and HAb-QD were thoroughly immobilized on the as-prepared platforms. Firstly, the samples of CBC and the AuNP-CBC, immobilized with the protein A at various conditions of pH and initial concentrations, were incubated with 100 µl HAb dispersed in a PBS buffer (0.01 M, pH 7.4) with a concentration of 16 µg/ml. The immobilization was accomplished by overnight incubation at 4 °C and 650 rpm. Then, the excess solution was discarded, and the samples were washed thrice with a PBS buffer (0.01 M, pH 7.4). Secondly, the 100 µl of the resultant and diluted HAb-QD, as described previously, were incubated with the finalized CBC-PA and AuNP-CBC-PA platforms. The same aforementioned conditions of the incubation and washing were applied here. Lastly, we pursued an optical test to examine the performance of the obtained nanopaper biomolecules. Briefly, 100 µl of HRP (0.02 g/l) dissolved in PBS buffer (0.01 M, pH 7.4) was added to the assembled nanopapers and made to interact with the immobilized HAb or HAb-QD for 1 h at room temperature and 650 rpm. Then, the uncaptured HRP was discarded and the samples washed three times with a PBS buffer (0.01 M, pH 7.4). The blank samples are considered to be the nanopaper immobilized with protein A and HRP. Afterward, 120 µl of TMB, the substrate for the enzymatic reaction of HRP, was added to the resultant platforms. After 30 s, the reaction was stopped by adding 120 µl of 2 M HCl while the solution color changed from blue to yellow. The optical density of the developed color was then measured at 460 nm using a TECAN spectroscopy device.

### Bacteria strain preparation

The bacterial dilutions were prepared each time before the experiments. The strain of *Escherichia coli* (DH5α) and *Staphylococcus aureus* (54–73) were provided by the Laboratory of Microbiology, Université de Montréal (Québec, Canada). *Escherichia coli* (ATCC 25922) was obtained from Cedarlane Co. (Burlington, Canada). The bacteria was picked up from the isolated colonies of a bacterial strain source in the agar plate followed by incubation in 5 ml sterile Luria–Bertani (LB) broth and grown overnight at 37 °C in an orbital shaker. Then, the bacterial suspension was separated from the LB broth by centrifugation at 8,000 rpm (g-force ≈ 6,007) for 3 min three times over and then resuspended in PBS buffer (0.01 M, pH 7.4). The stock solution of bacteria with the concentration of 1.5 × 10^8^ CFU mL^−1^ was defined by comparing with McFarland standard solution 0.5 (obtained from Thermo Fisher Scientific Inc., Ottawa, Canada). Lastly, the various concentrations of bacteria (1.5 × 10^8^–10 CFU mL^−1^) were obtained by tenfold serial dilution of the stock suspension in PBS buffer. The prepared dilutions were stored at 4 °C for later use.

### Preparation of the immunosensing platform for bacterial detection

The immunosensor platform for pathogen recognition was accomplished by the immobilization of EAb-QD or Alexa Fluor-EAb to the AuNP-CBC-PA.

The first step was to define the dependency of the quenching behavior of AuNP-CBCs on various densities of AuNP, where 100 µl of as-prepared EAb-QD was incubated with the samples of AuNP-CBCs-PA and CBCs-PA. This immobilization was conducted overnight at 4 °C and 650 rpm, as before. Next, after eliminating the excess reactants and washing the final assembled platform (three times, 100 µl PBS buffer, pH 7.4), its photoluminescent intensity was separately recorded for 16 distinct points (based on a standard pattern of a 4 × 4 filled square, excitation and emission wavelength at 350 and 525 nm, respectively) by the TECAN. The relative reduction of the QD fluorescence upon the attachment of EAb-QD was estimated as the ratio of its photoluminescent intensity on AuNP-CBC-PA to its photoluminescent intensity on CBC-PA. In the second step, 100 µl of as*-*dispersed *E. coli* (DH5α) in PBS buffer solution with a concentration of 10^6^ CFU mL^−1^ was then added to the platforms and incubated for 1 h at 25 °C. Next, the unattached bacteria were discarded without extra washing. The final fluorescent intensity (${I}_{f}$) of the immunosensor was then recorded at the same condition of the initial measurement, named ${(I}_{i})$. The overall performance of the fabricated immunosensor, presented in the of term quenching efficiency (${\varphi }_{F}$) of fluorophores, was evaluated by the following equation:

1 $${\varphi }_{F}=(1-\frac{{I}_{f}}{{I}_{i}})\times 100.$$

At the final step, subsequent to determining the optimized AuNP-CBC, with implementing the same conditions of the immobilization, EAb-QD or EAb-AF (100 µl dispersed in PBS buffer with a concentration of 16 µg/ml), the immunoassay platform was established. The assigned excitation and emission wavelength for those immunosensor platforms finalized with EAb-AF was 488 nm and 525 nm, respectively.

#### Evaluating the performance of the developed immunosensor for bacterial detection in the presence of a standard buffer

Prior to adding bacteria, the initial fluorescent intensity ${(I}_{i})$ of the platform was recorded with respect to the excitation and emission wavelength. The 100 µl of aforementioned dilutions of *E. coli* (DH5α) (10^8^,10^6^, 10^4^, 10^2^ and 10 CFU mL^−1^ dispersed in a PBS buffer) was then allocated to the immunosensor platforms (AuNP-CBC-PA immobilized with EAb-QD and EAb-AF) and incubated for 1 h at 25 °C. After removing the unattached bacteria, the ${I}_{f}$ was measured again, the quenching efficiency (${\varphi }_{F})$ for each sample was calculated by applying Eq. (1).

To examine the selectivity of the developed immunosensor (AuNP-CBC-PA immobilized with EAb-QD and EAb-AF), *E. coli* (ATCC 25922) and *S. aureus* were employed as the control bacteria. In this case, the diluted control bacteria in PBS buffer (10^8^,10^6^, 10^4^, 10^2^ and 10 CFU mL^−1^) were incubated under the same previous conditions with the immunosensor platforms, the fluorescent measurement, and the evaluation of ${\varphi }_{F}$ were proceeded as before.

The presented results in the following sections were calculated by means and standard deviation of 16 distinctive points measured on each sample, along with three parallel experimental samples to confirm the validity and reproducibility of the experiments. The blank sample for all experiments was considered as AuNP-CBC-PA immobilized with EAb-QD or EAb-AF, incubated with the suspension medium without bacteria.

#### Verifying the detection of Escherichia coli by using the developed immunosensor in real samples

As an example of the real sample matrix, poultry chicken and lettuce juice directly extracted from the chicken breast muscle and green leaves of lettuce were freshly provided from a butcher shop and grocery store in Montreal, Canada. Accordingly, 50 g of the chicken and lettuce leaves were separately mixed, each with 250 ml of PBS buffer in a blender. Afterward, the obtained homogenates were respectively filtered with Whatman filter NO. 1 to eliminate all large, suspended residues. Then, the bacteria were dispersed into the resulted filtrates to achieve the various concentrations by serial dilution of the stock solution.

The evaluation of *E. coli* (DH5α) detection with the concentrations of 10^6^, 10^4^, 10^2^,and 10 CFU mL^−1^ dispersed in the resulted filtrates for both platforms of AuNP-CBC-PA immobilized with EAb-QD and EAb-AF were conducted as previously performed for bacteria in the standard buffer.

## Results and discussion

The utilized bacterial cellulose (BC), with the appearance similar to a hydrogel sheet, was produced by acetic acid bacteria (e.g., *Acetobacter xylinum*) through oxidative fermentation of a medium^35^. The synthesized BC has a high degree of cellulose purity with a 3D structure composed of nanofiber with diameters of around 50–60 nm, resulting from the glucan chains linked strongly together by hydrogen bonds^36^. Besides, the abundant and natural hydroxyl groups on the cellulose structure offers plenty of functional groups for future modifications. All these features of BC assist the design of our proposed solid-based immunosensing array.

### Carboxylation of bacterial cellulose (CBC)

So, firstly, inducing carboxyl groups by oxidization of the BC nanofibers through TEMPO/NaBr/NaClO in aqueous solution transformed it into a nanopaper platform, which is simply able to link the biomolecules. Basically, TEMPO-mediated oxidation selectivity treats the primary hydroxyl groups located at C6 of the cellulose molecule^31^. As-resulted carboxyl group content on the CBC was estimated to be 0.5 mmol g^−1^ BC through the conductivity titration curve. These induced carboxyl groups allow a carboxyl-amine reaction for a robust bond between protein A and the surface of the CBC.

### In-situ synthesis of AuNP within CBC

**Figure 1:**
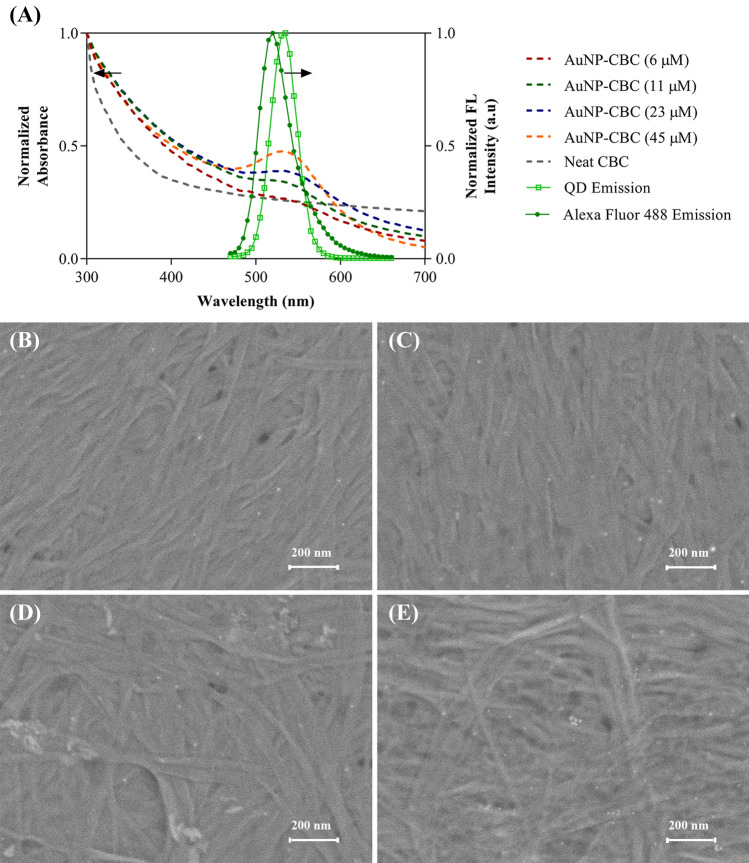
(**A**) UV–Vis absorbance spectra of the as-synthesized AuNP-CBCs with the various densities of AuNP by differing the precursor concentration from 6 to 45 µM, and their overlap with emission spectra of QD and Alexa Fluor 488 (excited at 350 and 488 nm, respectively), (**B**–**E**) SEM images from the AuNP-CBCs prepared by different concentrations of precursors, from the low to high concentrations, respectively.

Secondly, by embedding the AuNP within the CBC, we obtained a plasmonic nanopaper and solid-based platform with a strong quenching behavior applicable to absorb energy transfer from the fluorophores. Through the Turkevich method, Au ions were reduced, capped by citrate ions, and led to the in-situ synthesized AuNP-CBC. As observed, the density of the AuNPs was adjustable by changing the concentration of the Au precursor (by using 6, 11, 23, and 45 µM of hydrogen tetrachloroaurate (III) hydrate) that caused the color alteration of CBC pieces from colorless to light or saturated maroon. Compared with the plasmonic properties of AuNP, similar behavior was observed in the UV–Vis absorbance spectra of AuNP-CBCs with the absorbance peak around 535 nm (Fig. 1A) in which reducing the density of synthesized AuNPs led to a decrease in the intensity of the absorbance peak making it wider. Precisely, the electron microscopy imaging, as shown in Fig. 1B–E, confirmed the formation and uniform distribution of AuNPs within the CBC nanofibrils. However, for the lower concentrations of the precursor (6 and 11 µM) it is barely recognizable. Also, the evaluated carboxyl group content of AuNP-CBCs (see “Experimental section”) was reduced between 5 and 10% (from low to high density of AuNP-CBCs) in comparison with the plain CBC. This could be attributed to either hindrance of the carboxyl groups by the AuNPs or their contribution to the formation of nanoparticles. The obtained nanopapers inherently took advantage of the CBC with its carboxyl groups, plasmonic properties of AuNP and their capability of absorbing the energy transfer from a fluorophore, as they showed an overlap with the emission spectra of quantum dot nanoparticles and Alexa Fluor 488, exhibited in Fig. 1A.

### Protein A immobilization on the nanopaper platform and its characterization

Protein A is a recombinant protein that can bind strongly to the Fc region of an antibody, and therefore, a well-oriented antibody configuration can be established through their affinity binding^37,38^. Here, the protein A was immobilized on the surface of the bare CBC and AuNP-CBC through EDC/Sulfo-NHS chemistry by covalent binding between amino groups of the protein A and the activated carboxyl groups. The performance of the bound protein A was assessed by its interaction with the model antibody anti-HRP (HAb). Since protein A plays a significant role in the orientation of the conjugated antibody, it would affect the final sensitivity of the immunosensor. Therefore, having a well-established antibody on the surface of the nanopaper is an essential and primary step in the preparation of a high efficient immunosensor. The suggested strategy allowed us to improve our understanding of the integrity of the bioassay platform. Previous studies have explored the direct correlation of properly immobilized protein A with the number of bound antibodies that decisively influenced their interaction with the respective antigen^37,39^. In fact, the number of captured analytes (e.g., HRP) can directly correspond to the quantity and placement of the respective antibodies. On the other hand, estimating the HRP activity after interacting with HAb by optical density can directly lead us to the position of antibodies linked to protein A and, therefore, the situation of the protein A on the nanopaper platform.

First, the study of the pH effect on the immobilization of protein A on the surface of the plain CBC, as presented in Supplementary Fig. S1A showed a higher response of HRP captured by HAb, corresponding to a pH of 4.1. Moreover, the Bradford assay analysis confirmed a greater quantity of the immobilized protein A at this pH in comparison with those immobilized at a pH of 7.4. As displayed in Supplementary Table S1, the evaluated amount of the immobilized protein A is 3.96 ± 0.04 and 2.62 ± 0.01 µg carrying out at pH 4.1 and 7.4, respectively. This pH of immobilization is very close to the isoelectric point (PI) of protein A (4.65, supplier’s data), which can impose a charge distribution on its amino group, different from the pH of 7.4. This fact can relatively control the orientation of the deposited protein A, which can significantly influence the antibody linkage. Secondly, our experiments revealed the capacity of the CBC to bind protein A (see Supplementary Fig. S1B). However, the maximum amount protein A bonded to the CBC was measured about 11.76 ± 0.14 µg (presented in Supplementary Table S1), the estimated performance of HRP was approximately equal to 3.95 ± 0.05 µg of the immobilized protein A. It was also noted that if the immobilized protein A was reduced to 1.31 ± 0.09 µg (67% less protein A in comparison with the previous amount), the HRP activity decreased just by 30%, indicating a nonlinear correlation. These results may suggest that a strong presence of protein A might hinder its available active sites preventing appropriate bonding for the antibodies. On the other hand, the increase in the number of the protein A on the CBC nanofibers caused an increase in the number of linked antibodies on protein A. However, as the consequence of this aggregation, the active sites of HAb might be compromised, and therefore, are prevented from being well accessible to HRP. Given that the strategy of the protein A immobilization on the surface of the nanopaper, which affects its orientation and hindrance, its assessment for each individual platform was necessary to increase the sensitivity of the final developed bioassay.

As-determined highly efficient conditions of protein A immobilization on CBC (immobilization pH 4.1 with the initial concentration of 0.1 mg/ml) were assigned then after for its immobilization to the AuNP-CBC surface. The Bradford assay analysis indicated that 2.77 ± 0.09, 3.14 ± 0.07, 3.78 ± 0.06, 3.86 ± 0.09 µg of the protein A was bonded to carboxylated AuNP-CBCs (from the high to low density of AuNP-CBCs), respectively, as presented in the Supplementary Table S1. This result is in agreement with the outcome of conductimetry titration, as it previously revealed a reduction in the content of carboxyl groups after the synthesis of AuNPs. So, the decrease of available functional groups led to a slight reduction in immobilized protein A. However, as noted in Supplementary Fig. S2A, the comparison between the activity of HAb linked to the plain CBC-PA and AuNP-CBC-PA (with the precursor of 23 µM) revealed a negligible difference that shows this slight reduction in the amount of immobilized protein A did not considerably influence the HAb binding. Likeness, the HAb-QD bonded to both platforms had similar activity behavior, although its overall functionality was decreased in comparison with the unlabeled HAb. It is worth mentioning that the trivial difference between the observed HRP optical density captured by the HAb-QD and that captured by the bare HAb (see Supplementary Fig. S2A) may be attributed to the presence of QD. According to the size of the conjugated QD (3.54 ± 0.52 nm, shown in Supplementary Fig. S3) and its position in the antibody, it may insignificantly impact the ability of the antibody to bind to the antigen or link to protein A. Despite this trial effect, the overall performance of the developed bioassay for HRP recognition was estimated through the change of intensity of QD fluorescence. As Supplementary Fig. S2B presents, a quenching efficiency of 20% was assessed by the interaction of HRP and HAb-QD. It confirmed that the conformational change of HAb-QD resulted in the alteration of the distance between QD and AuNP-CBC. The change in the antibody structure is notably affected by the type and size of antigen; as binding to a larger antigen, such as bacteria, can induce more changes in the constant and variable domains of the antibody^28^.

### Preparation of the immunosensing platform for pathogen detection

**Figure 2:**
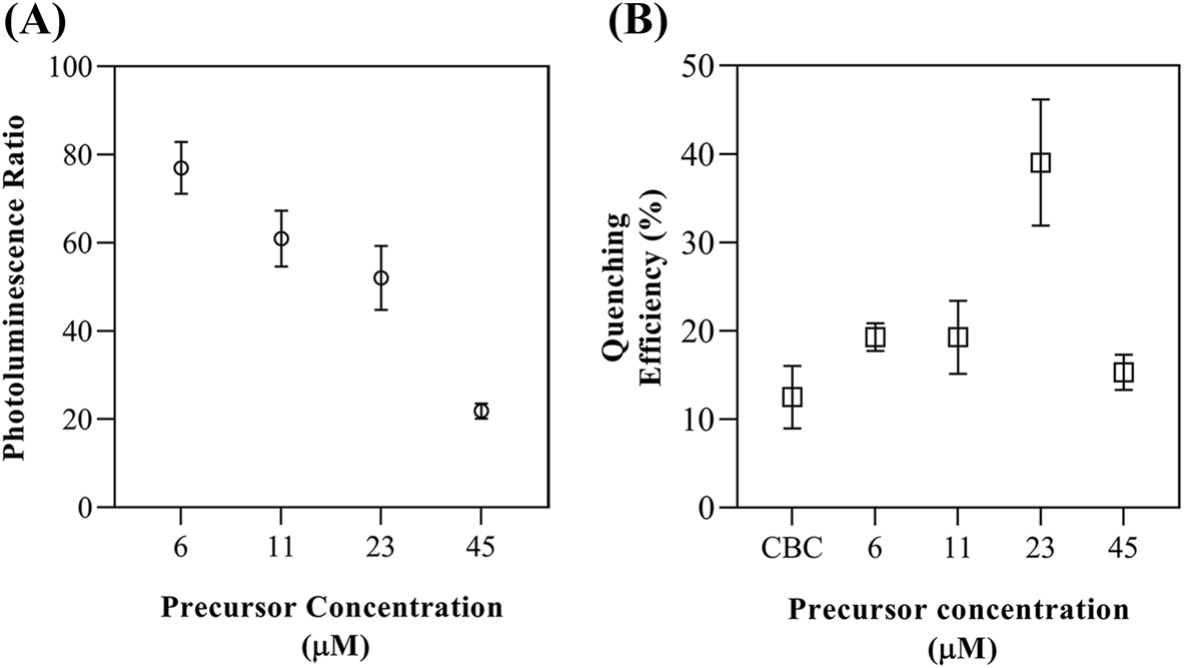
(**A**) The photoluminescence ratio of EAb-QD after its linkage to the AuNP-CBC-PA with various densities to CBC-PA and, (**B**) the quenching efficiency of EAb-QD linked to AuNP-CBC-PA and CBC-PA after capturing *E. coli* (Dh5α).

Beyond the role of the protein A for establishing oriented and active antibodies, AuNP should be considered as an important element for revealing the sensing response. So, it is crucial to estimate the effect of AuNP density on the quenching of QD photoluminescence bonded EAb. In this respect, the photoluminescence of EAb-QD after its link to the AuNP-CBCs-PA platforms with various densities of gold nanoparticles was thoroughly compared with CBC-PA. As Fig. 2A demonstrates the ratio of quenching, the highest density of AuNP-CBCs-PA (precursor concentration of 45 µM) reduced the QD fluorescent to 20%. By reducing the precursor concentrations to 23, 11, and 6 µM, the fluorescence of QD were dramatically increased up to 80%, whereas, as noted in Fig. 2A, the photoluminescence of QD has non-linearly corresponded to the density of AuNP within CBCs. In fact, the rate of energy transfer from a donor to an acceptor increases by the enhancement of acceptor molecules^40^. These AuNP-CBCs-PA were therefore evaluated in their ability to quench the fluorophore after bacterial attachment. The AuNP-CBC with the initial precursor concentration of 45 µM wasn’t considered a proper platform due to its potent effect on the quenching of EAb-QD. As illustrated in Fig. 2B, for detecting 10^–6^ (CFU mL^−1^) *E. coli* (DH5α), the maximum quenching efficiency around 40%, was achieved through AuNP-CBC-PA/EAb-QD with a precursor concentration of 23 µM.

As we noted, there is an initial and secondary quenching phenomenon, with the first happening after the linkage of EAb-QD to the platform of immunosensor (AuNP-CBCs-PA) and the second resulting from the attachment of bacteria to the immunosensor. The high initial quenching related to the high density of AuNP-CBC (e.g., precursor 45 µM) would not allow monitoring of the fluorophore response after capturing the bacteria. Conversely, the low-density AuNP-CBC could not act efficiently to reveal the bacteria-antibody interaction in terms of changes in the photoluminescence. As such, this assessment provided a general understanding from the effect of synthesized AuNP into CBC regarding quenching processes and allowed us to find out the most effective platform for the detection of bacteria, that is the platform synthesized with 23 µM precursor of gold solution precursor.

### Bacterial recognition in the presence of a standard buffer

**Figure 3:**
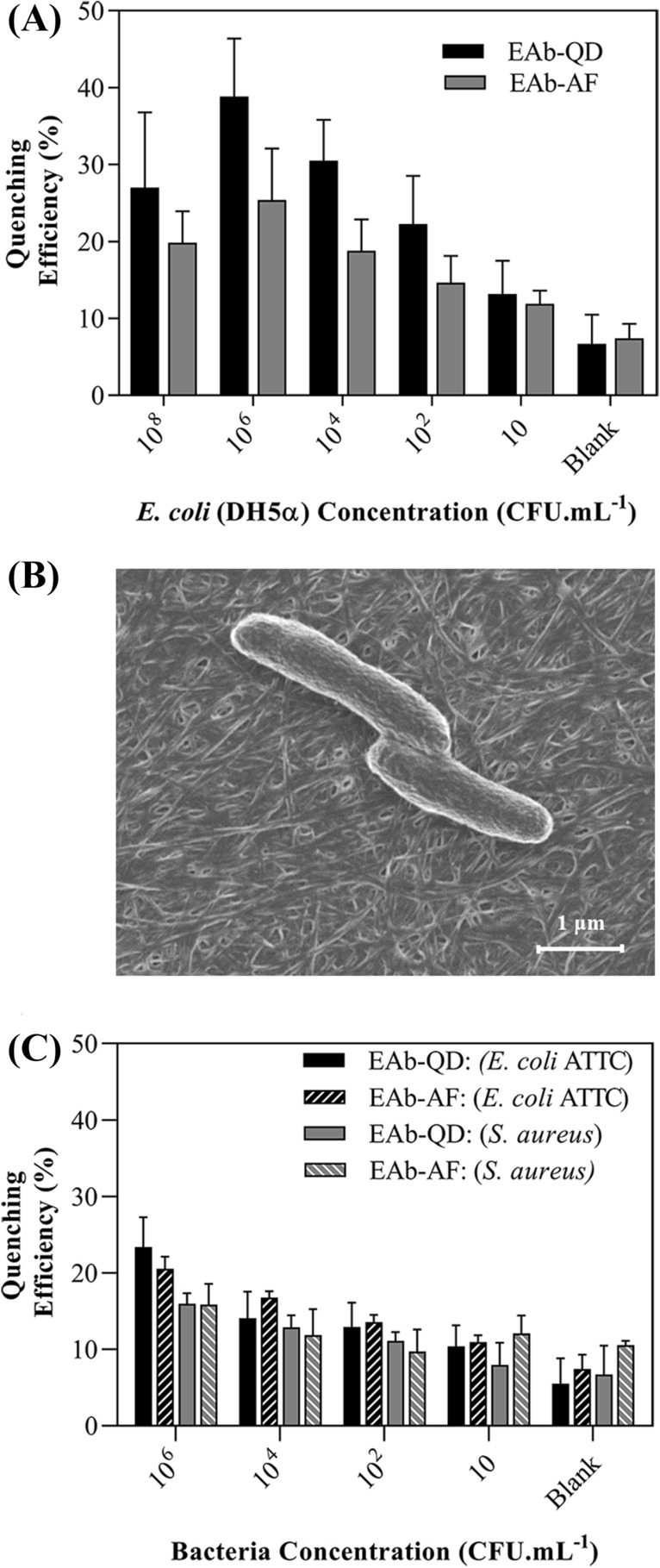
Evaluation of the immunosensor fabricated by AuNP-CBC-PA/EAb-QD and AuNP-CBC-PA/EAb-AF (**A**) the sensitivity of the proposed immunosensors toward various concentrations of *E. coli* (DH5α), (**B**) SEM image of the captured *E. coli* (DH5α) on AuNP-CBC-PA/EAb-QD and (**B**) their specificity and selectivity against different concentrations of *E. coli* (ATCC 25922) and *S. aureus*.

In this step, the proficiency of the proposed immunosensor was first examined for various concentrations of *E. coli* (DH5α), diluted from 10^8^ to 10 CFU mL^−1^ in a standard buffer, as presented in Fig. 3A. Interestingly, the evaluated quenching efficiency (${\varphi }_{F})$ showed a smaller value for the concentrations of 10^8^ CFU mL^−1^ rather than concentrations of 10^6^ CFU mL^−1^, which may define the highest capacity of this immunosensor for bacterial recognition that is 10^6^ CFU mL^−1^. By lowering the concentration of bacteria from 10^4^ to 10 CFU mL^−1^, the evaluated quenching efficiency reduced to a closer value of the blank sample. For the blank sample, there should not be any decrease in the QD photoluminescent, but the trivial reduction may be induced by either removing the extra EAb-QD or its slight detaching from protein A during the time of incubation. Figure 3B illustrates the SEM images from the *E. coli* (DH5α) (fixed with 2.5% glutaraldehyde) after its capture by the EAb-QD on the AuNP-CBC-PA platform.

To prove whether the change of fluorophore can influence the performance of the proposed scenario, the immunosensor employed EAb-AF [antibody against *E. coli* (DH5α) conjugated Alexa Fluor 488] was tested against the mentioned concentrations of *E. coli* (DH5α). As shown in Fig. 3A, although this configuration perfectly functioned to detect the presence of bacteria, a diminished value in the quenching efficiency is clear in comparison with the response of EAb-QD. It may be attributed from the positions of Alexa Fluor on the structure of the antibodies and differences in both shape and composition. The performance of these immunosensors was compared through the estimation in their limit of detection (LOD), which is the quenching efficiency of the blank sample plus three times its standard deviation. As resulted, the threshold of LODs for both immunosensors was interestingly determined to be as low as 50 CFU mL^−1^, confirming the sensitivity of this nanopaper-based immunosensor.

Furthermore, the specificity and selectivity of the developed immunosensor were thoroughly evaluated by spiking various concentrations of bacteria such as *E. coli* (ATCC 25922) and *Staphylococcus aureus* (*S. aureus*) (see Fig. 3C). The estimated quenching efficiency through AuNP-CBC-PA/EAb-QD and CBC-PA/EAb-AF for the highest concentration of *S. aureus* (10^6^ CFU mL^−1^) were within the range of the calculated LOD of *E. coli* (DH5α). Meanwhile, by decreasing the concentration of *S. aureus* (from 10^4^ to 10^2^ CFU mL^−1^), the respective quenching efficiencies declined under the LOD threshold. In addition, both developed immunosensors, AuNP-CBC-PA/EAb-QD and AuNP-CBC-PA/EAb-AF, slightly responded against *E. coli* (ATCC 25922) due to the partial cross-reactivity, since both *E. coli* strains may indicate similar targeting sites, positioned on the outer membrane^41^. However, the cell wall compositions can be varied likely within a single genus and various strains of bacteria, driving from parameters such as culturing conditions and growth phases^42,43^.

### The performance of the immunosensor for bacterial detection in real samples

**Figure 4:**
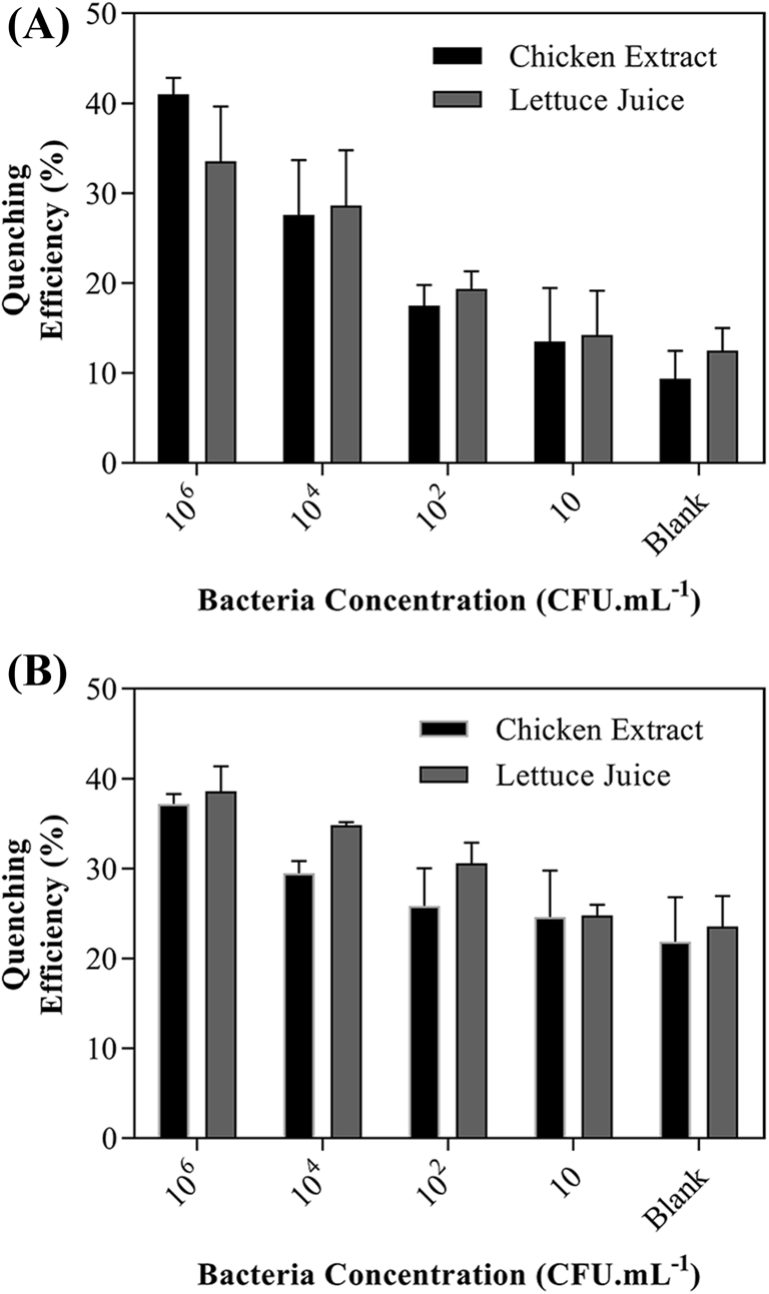
The performance of immunosensors AuNP-CBC-PA immobilized with (**A**) EAb-QD and (**B**) EAb-AF toward various concentrations of *E. coli* (DH5α) dispersed in the chicken extract and the lettuce juice.

Although the presented results obtained from the functionality of immunosensors in the standard buffer are a determined proof of a state-of-the-art approach, the validity of the immunosensors was also proven through its ability to detect bacteria in real or semi-real conditions. In this respect, the dispersed *E. coli* (DH5α) bacteria in extracts of chicken and lettuce juice with various concentrations were spiked into both immunosensors developed with EAb-QD and EAb-AF, as Fig. 4A,B present the performance of each immunosensor platform. By comparison, it is obvious that the immunosensor fabricated with EAb-QD has a higher performance in detecting bacteria. As estimated, the threshold of LOD for the immunosensor decorated with EAb-QD in both matrices, chicken extract, and lettuce juice was around ~ 10^2^ CFU mL^−1^. While, the immunosensor with EAb-AF indicated a poor performance with a LOD of 10^4^ and 10^6^ CFU mL^−1^ for recognizing bacteria dispersed in the chicken extract and lettuce juice, respectively. This performance may arise from an undesired interaction between the Alexa Fluor and the ingredient available in the real matrices, nevertheless the appropriate reason is not fully known to us. Hence, the immunosensor-based QD demonstrated remarkable reliability to be utilized in a contaminated real matrix.

The major advantage of our designed immunosensor is the employment of the plasmonic nanopaper to address the benefits of a flexible, sustainable substrate that can overcome the disadvantages of a single quencher nanoparticle such as the aggregation. We also relied on the inherent properties of an antibody that dismisses the need of a secondary labeled antibody to achieve the desired performance, which is a challenge in most of the developed bioassays and may limit their application for on-site detection. Although this approach was proposed as a proof-of-concept for a novel, solid, nanopaper-based immunoassay, it exhibited appropriate sensitivity toward the recognition of pathogenic bacteria.

## Conclusion

In this study, we succeeded in fabricating a novel, versatile immunosensor on a nanopaper platform for the detection of *E. coli*. The presence of bacteria was revealed by a reduction in the photoluminescence of the fluorophore-labeled (QD or Alexa Fluor 488) antibody. Since the energy transfer occurred between the fluorophore and plasmonic nanopaper (CBC embedded with gold nanoparticles (AuNP-CBC) served as the acceptor) upon the interaction of the antibody and bacteria, depending on the conformational change in the structure of the antibody. Through this technique, *E. coli* (DH5α) was recognized as low as 50 CFU mL^−1^ in the standard buffer. Examining the feasibility of the immunosensor in real matrix samples, employed antibody conjugated QD, exhibited a threshold of LOD around ~ 10^2^ CFU mL^−1^, while the immunosensor fabricated with antibody conjugated Alexa Fluor 488 poorly functioned. This immunosensor identified the bacteria directly without employing a secondary antibody or any other biomolecule. This immunosensor was presented as proof of concept, and it has the potential to be improved for multiple detections with respect to the unique features of AuNP-CBC and QDs. Additionally, the simplicity, selectivity, and specificity of the proposed immunosensor allow its potential to be used as a portable analytical device in medical, food, and environmental industries.

## Supplementary information

Supplementary Information.
