# Supplementary material for: Toward a nanopaper-based and solid phase immunoassay using FRET for the rapid detection of bacteria
Source: Sci Rep. 2020 Sep 1;10:14367. doi: 10.1038/s41598-020-71285-3 (PMC7463021; doi:10.1038/s41598-020-71285-3)
Supplement: Supplementary file 1 — Supplementary Information. [file 41598_2020_71285_MOESM1_ESM.docx]

Toward a Nanopaper-Based and Solid Phase Immunoassay Using FRET for the Rapid Detection of Bacteria

Bentolhoda Heli, Abdellah Ajji^*^

3SPack, CREPEC, Département de génie chimique École Polytechnique de Montréal, Montréal, Québec, Canada.


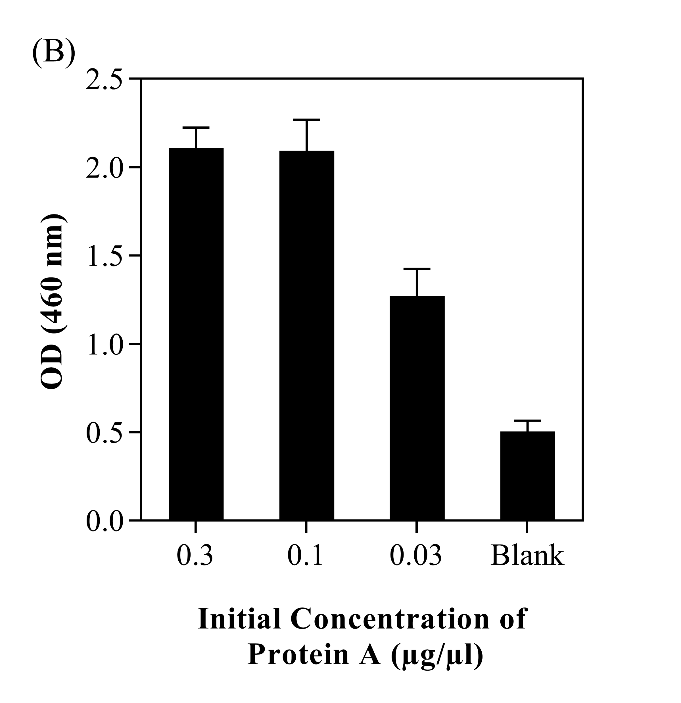

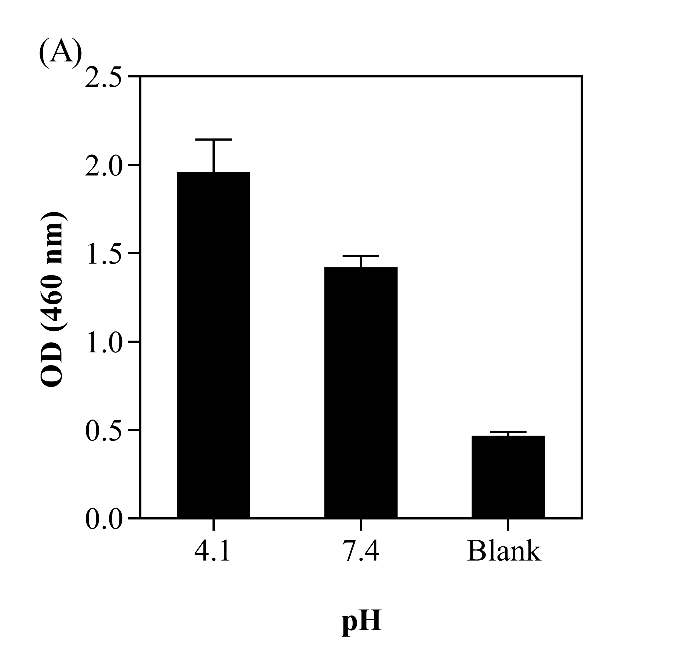


Figure S1. The optical density recorded at 460 nm as a result of the enzymatic reaction of HRP and TMB. It presents the situation of the protein A immobilized on the surface of CBC by the interaction between the HAb linked to protein A and then capturing HRP. (A) The effect of pH on the immobilization of protein A, (B) the effect of the initial amount of protein A on its immobilization.^1^


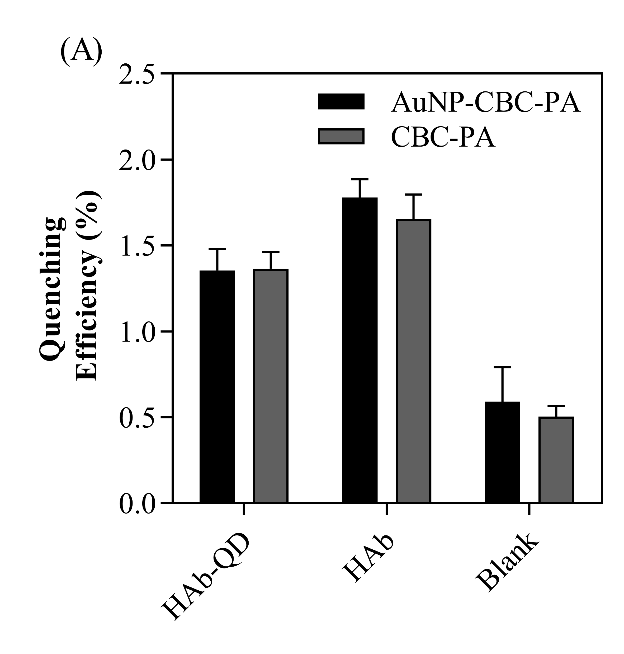


Figure S2. (A) Estimated optical density of HRP response captured by HAb and HAb-QD, which are linked to protein A in two different platforms, AuNP-CBC and neat CBC, (B) quenching efficiency evaluated by the capture of HRP to HAb-QD which is linked to protein A on the surface of AuNP-CBC.^1^

Table S1. A comparison between the amount of immobilized protein A in different condition and various platforms. These amounts are estimated through the Bradford assay analysis.

| Conditions  Platforms | Initial Amount of Protein A (µg) | Immobilized Amount of Protein A (µg) | pH of immobilization |
| --- | --- | --- | --- |
| CBC | 4 | 2.62±0.01 | 7.4 |
|  | 4 | 3.96±0.04 | 4.1 |
|  | 13.2 | 11.76±0.14 | 4.1 |
|  | 4.4 | 4.29±0.05 | 4.1 |
|  | 1.32 | 1.31±0.09 | 4.1 |
| AuNP-CBC (Precursor: 6 µM) | 4 | 3.86±0.09 | 4.1 |
| AuNP-CBC (Precursor: 11 µM) | 4 | 3.78±0.06 | 4.1 |
| AuNP-CBC (Precursor: 23 µM) | 4 | 3.14±0.07 | 4.1 |
| AuNP-CBC (Precursor: 45 µM) | 4 | 2.77±0.09 | 4.1 |

B

A


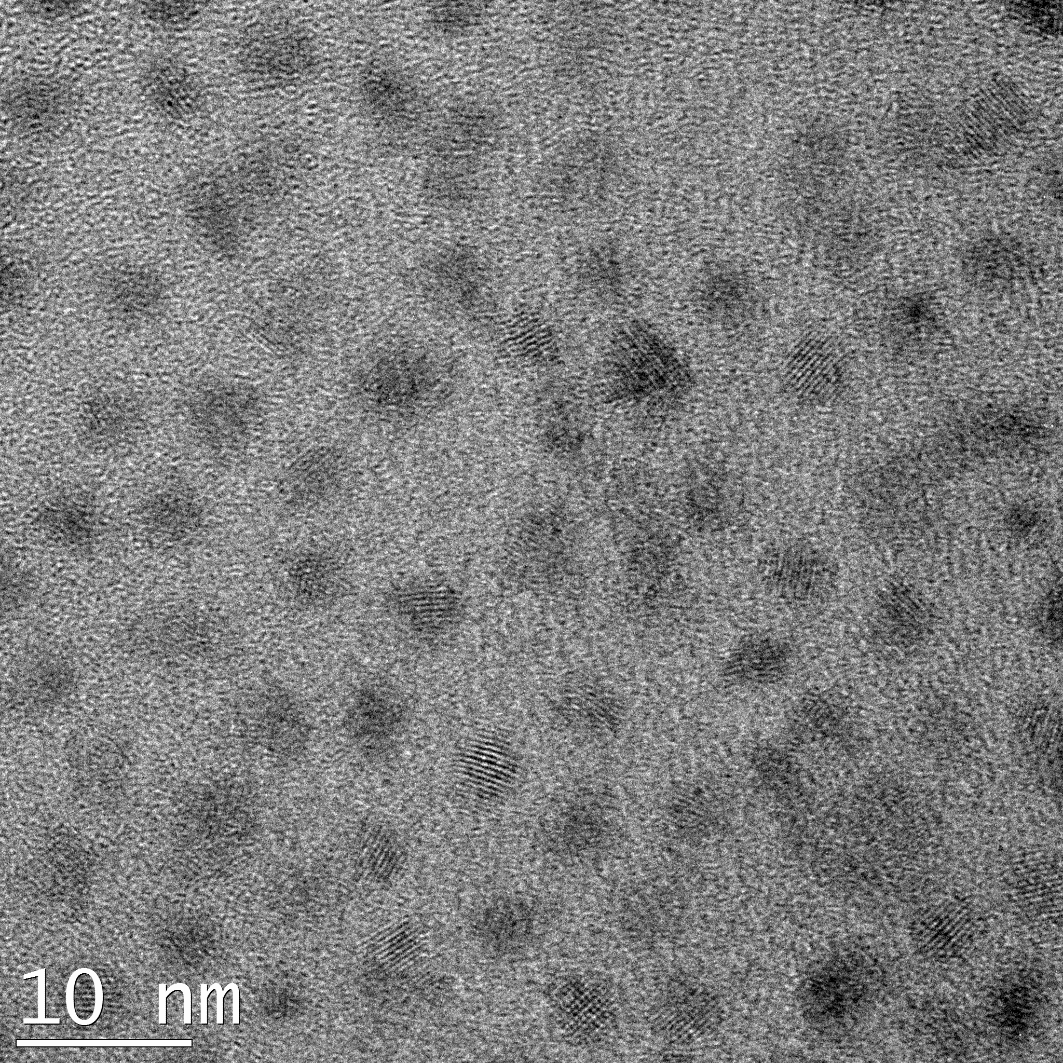

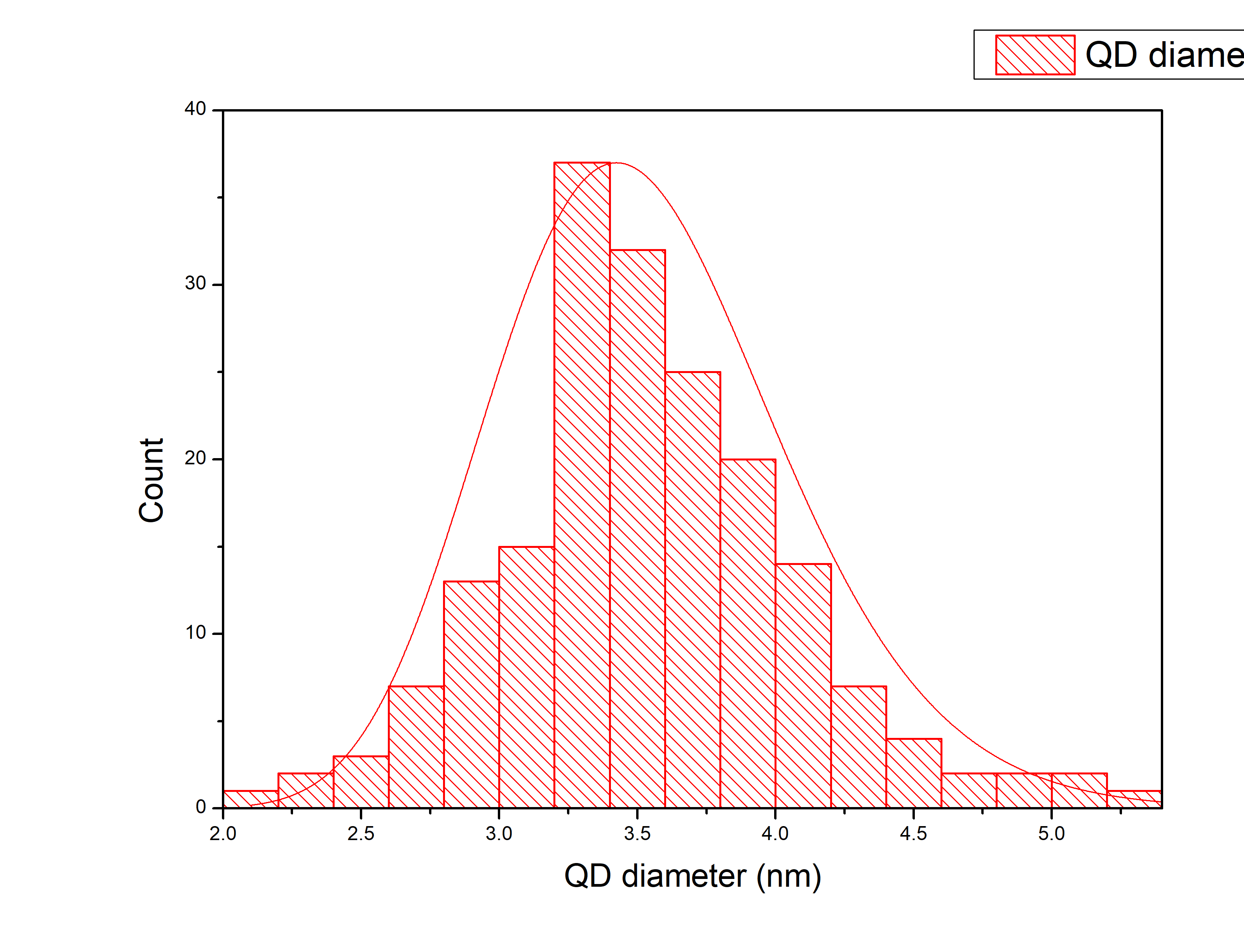


Figure S3. (A) TEM image of QD which was conjugated to antibody and (B) The size distribution of QD obtained by analyzing the diameter of 200 individual QD particles.^1^

Reference:

1 Heli, B. *Bacterial cellulose supported sensor for bacteria and gas detection.* Ph.D. thesis, Polytechnique Montréal, (2017).
